# The Prognostic Value of Adjuvant Chemotherapy in Colon Cancer With Solitary Tumor Deposit

**DOI:** 10.3389/fonc.2022.916091

**Published:** 2022-07-13

**Authors:** Qiangkang Lin, Huizhen Zhou, Si Shi, Jixu Lin, Wangxin Yan

**Affiliations:** Department of Colorectal and Anal Surgery, The Third Affiliated Hospital of Shanghai University, Wenzhou People’s Hospital, Wenzhou No. 3 Clinical Institute Affiliated to Wenzhou Medical University, Wenzhou, China

**Keywords:** adjuvant chemotherapy, colon cancer, solitary, tumor deposit, survival

## Abstract

**Purpose:**

The aim of this study is to investigate the survival benefit of adjuvant chemotherapy in patients with colon cancer with the solitary tumor deposit (TD).

**Methods:**

The primary study outcomes used in this study were colon cancer–specific survival (CSS) and overall survival (OS). The differences of the distribution of categorical variables in patients with colon cancer with the solitary TD according to adjuvant chemotherapy administration were tested using the Pearson’s chi-square test. The Kaplan–Meier method was utilized to evaluate CSS and OS. Hazard ratio (HR) and 95% confidence interval (CI) were calculated on the basis of Cox regression models to assess the prognostic value of different demographic and clinicopathological characteristics.

**Results:**

A total of 877 patients with TanyN1cM0 colon cancer with solitary TD were identified in our analysis. It was found that OS (75.4% vs. 42.8% for 5-year OS rate, p < 0.001) and CSS (82.9% vs. 69.3% for 5-year CSS rate, p < 0.001) of patients with colon cancer with adjuvant chemotherapy administration were significantly better than those without adjuvant chemotherapy administration. Multivariate Cox survival analyses revealed that the overall and colon cancer–specific mortality risks of patients with adjuvant chemotherapy administration were decreased by 64.4% (HR = 0.356, 95% CI = 0.265–0.479, p < 0.001) and 57.4% (HR = 0.426, 95% CI = 0.286–0.634, p < 0.001) compared with those without adjuvant chemotherapy administration, respectively.

**Conclusions:**

Adjuvant chemotherapy administration could significantly improve OS and CSS in patients with colon cancer with the solitary TD. This is the first study to investigate and demonstrate the survival benefit of adjuvant chemotherapy in patients with colon cancer with the solitary TD.

## Introduction

Colorectal cancer is now the third most common cancer in the world and the second most common cause of cancer death (1). The tumor, node, metastasis (TNM) staging is the most generally used system for the diagnosis and treatment of colorectal cancer, and it has been continuously developed with the continuous progress of tumor treatment; the latest eight edition of the TNM staging system was published in 2017 (2, 3).

In the eighth edition of TNM staging, tumor deposit (TD) is defined as tumor foci located in the pericolonic or perirectal fat, away from the tumor invasion front, in the lymphatic drainage area of ​​the tumor, with no identifiable residual lymph node tissue. In addition, the presence of TD is classified as N1c in the absence of lymph node metastasis (LNM) and TD has no impact on tumor stage in the presence of LNM. Previous studies have shown that TD is an independent and strong prognostic factor in patients with colorectal cancer after surgery, regardless of lymph node status (4–6). More importantly, many studies have shown that the number of TDs is independently associated with poor outcomes in patients with colorectal cancer and suggested adding TDs to the number of LNMs (7–9). A meta-analysis found that TD was a stronger prognostic factor than LNM or extramural vascular invasion for patients with colorectal cancer with liver, lung, and peritoneal metastases (10). In addition, TD is also significantly associated with higher local recurrence and worse prognosis in patients diagnosed with rectal adenocarcinoma (11, 12).

To date, however, no studies have evaluated the role of adjuvant chemotherapy in patients with colon cancer with solitary TD although adjuvant chemotherapy is routinely used in patients with N1c colorectal cancer. Recently, Korean researchers retrospectively analyzed the effect of adjuvant chemotherapy in 281 patients with colon cancer and found that adjuvant chemotherapy did not provide clear survival benefit for patients with colon cancer with solitary LNM (13). Therefore, it is reasonable to question the survival benefit of adjuvant chemotherapy in patients with colon cancer with solitary TD.

## Material and Methods

### Patients’ Selection and Cohorts

Patients with solitary TD are a very small group of people in colon cancer; therefore, a large cancer database is utilized in this study. The National Cancer Institute’s Surveillance, Epidemiology, and End Results (SEER) database is the largest cancer database in the United States, containing information on patient’s age, sex, tumor stage, tumor site, tumor grade, treatment, and prognosis of malignant tumors in 18 population-based cancer registries, covering about one-third of the population in the country. We then extract the patient data from the SEER database (https://seer.cancer.gov). Because patient identification information has been removed in this database, ethics committee approval and patient informed consent are not required in this study.

**Figure 1:**
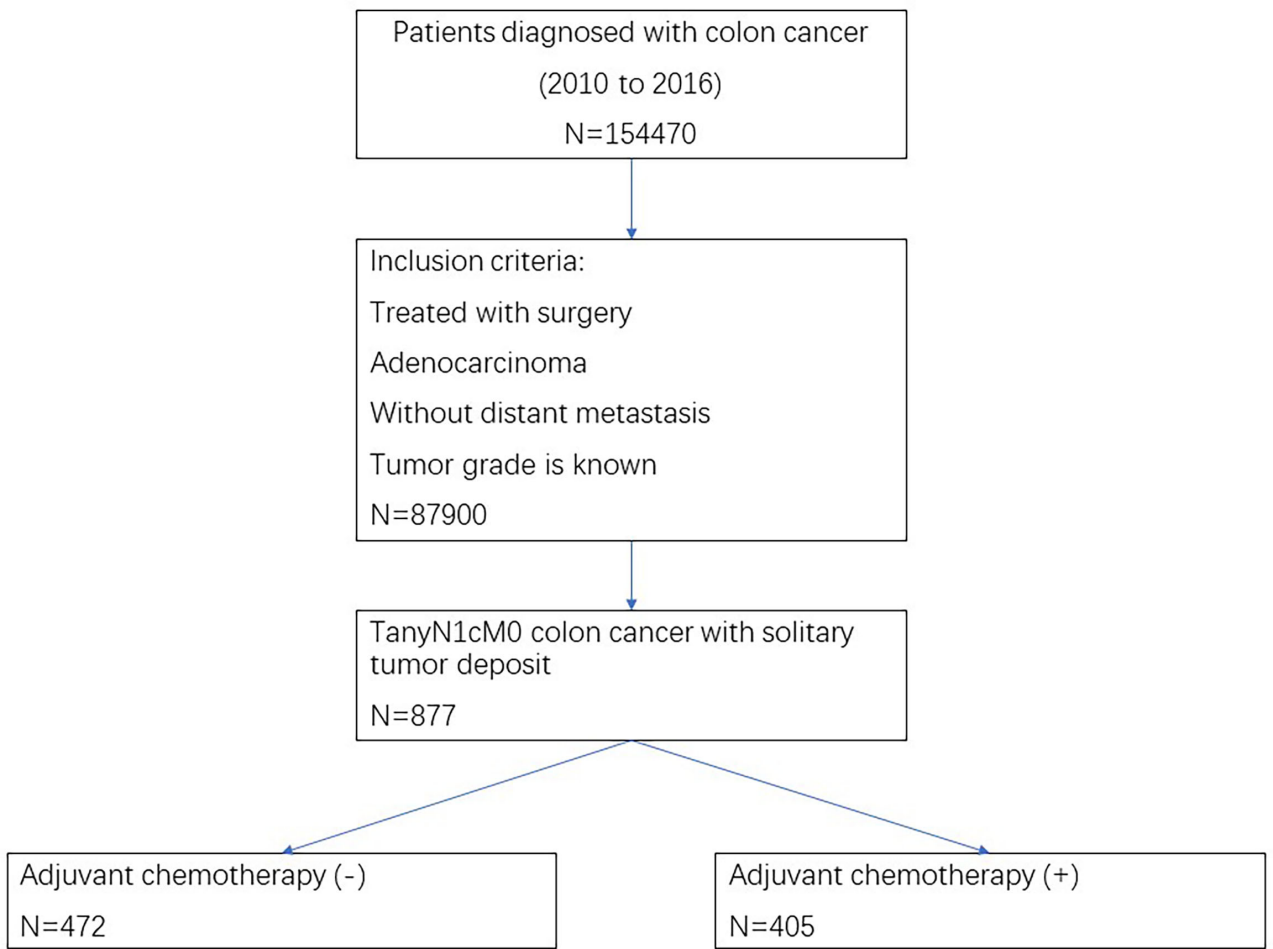
Flow chart of case selection.

As shown in **Figure 1**, we first selected patients diagnosed with colon cancer in the cancer database from 2010 to 2016, and patients who met the following criteria were further confirmed: treated with surgery, adenocarcinoma, without distant metastasis, and tumor grade is known. Finally, patients with TanyN1cM0 colon cancer with the solitary TD were selected for the analysis of this study.

### Statistical Analysis

The primary study outcomes used in this study were colon cancer–specific survival (CSS) and overall survival (OS). CSS and OS were defined as the time between the cancer diagnosis and death due to colon cancer and death from any cause, respectively. Patients’ demographic and clinicopathological characteristics were included as follows: T staging (T1, T2, T3, and T4), age at diagnosis (≤65 and >65 years old), gender (male and female), tumor location [right-sided colon (from caecum to transverse colon) and left-sided colon (from splenic flexure to rectosigmoid junction)], and tumor grade (grade I/II and grade III/IV).

The differences of the distribution of categorical variables in patients with colon cancer with the solitary TD according to adjuvant chemotherapy administration were tested using the Pearson’s chi-square test. The Kaplan–Meier method was used to evaluate CSS and OS. The log-rank test was also carried out to assess whether the differences of CSS or OS rates between subgroups were statistically significant. Hazard ratio (HR) and 95% confidence interval (CI) were calculated on the basis of Cox regression models to assess the prognostic value of different demographic and clinicopathological characteristics. Statistically significant levels were two-tailed, and a p-value less than 0.05 was considered significant. Statistical analyses were conducted using the Statistical Package for the Social Sciences version 23.0 software (IBM Corporation, Chicago, IL, USA).

## Results

### Patient Characteristics

As shown in **Figure 1**, a total of 877 patients with TanyN1cM0 colon cancer with solitary TD were identified in our analysis, among which 405 (46.2%) patients received adjuvant chemotherapy and 472 (53.8%) patients did not receive adjuvant chemotherapy; 29 (3.3%) patients were diagnosed with stage T1, 80 (9.1%) patients were diagnosed with stage T2, 567 (64.7%) patients were diagnosed with stage T3, and 201 (22.9%) patients were diagnosed with stage T4; 332 (37.9%) patients were less than 65 years old, and 545 (62.1%) patients were more than 65 years old; 453 (51.7%) patients were male, and 424 (48.3%) patients were female; 489 (55.8%) patients were diagnosed with right-sided colon cancer, and 388 (44.2%) patients were diagnosed with left-sided colon cancer; 727 (82.9%) patients were diagnosed with grade I/II colon cancer, and 150 (17.1%) patients were diagnosed with grade III/IV colon cancer.

Patients’ demographic and clinicopathological characteristics according to adjuvant chemotherapy administration were shown in **Table 1**. There were no differences in T staging, gender, tumor grade, and number of lymph nodes examined between the two subgroups. Younger age (56.5% vs. 21.8% for ≤ 65 years old, p < 0.001) and left-sided colon cancer (48.6% vs. 40.5% for left-sided colon cancer, p < 0.015) were more likely to be associated with the adjuvant chemotherapy administration.

**Table 1:** Clinicopathologic characteristics of colon cancer with solitary tumor deposit according to adjuvant chemotherapy administration.

Variables	Adjuvant Chemotherapy (−) (n = 472)	Adjuvant Chemotherapy (+) (n = 405)	*P-*value
**T staging**			0.111
** T1**	10 (2.1%)	19 (4.7%)	
** T2**	44 (9.3%)	36 (8.9%)	
** T3**	316 (66.9%)	251 (62.0%)	
** T4**	102 (21.6%)	99 (24.4%)	
**Age at diagnosis (years)**			<0.001
** ≤65**	103 (21.8%)	229 (56.5%)	
** >65**	369 (78.2%)	176 (43.5%)	
**Gender**			0.807
**Male**	242 (51.3%)	211 (52.1%)	
**Female**	230 (48.7%)	194 (47.9%)	
**Tumor location**			0.015
** Right-sided colon**	281 (59.5%)	208 (51.4%)	
** Left-sided colon**	191 (40.5%)	197 (48.6%)	
**Grade**			0.259
** I/II**	385 (81.6%)	342 (84.4%)	
** III/IV**	87 (18.4%)	63 (15.6%)	
**No. of lymph nodes examined**			0.056
** <12**	76 (16.1%)	47 (11.6%)	
** ≥12**	396 (83.9%)	358 (88.4%)	

### Survival Benefit Conferred by Adjuvant Chemotherapy in All Patients With Colon Cancer With Solitary Tumor Deposit

**Figure 2:**
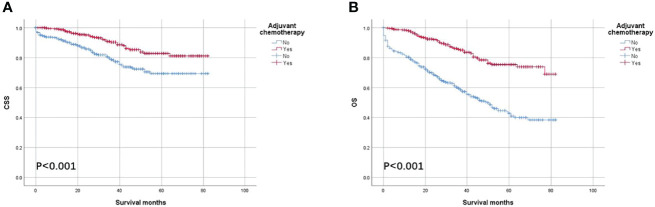
Kaplan–Meier survival curves comparing **(A)** CSS and **(B)** OS according to the use of adjuvant chemotherapy in all patients with colon cancer with solitary tumor deposit.

As shown in **Figure 2**, the CSS and OS curves of patients with colon cancer with the solitary TD were generated using the Kaplan–Meier method. It was found that OS of patients with colon cancer with the solitary TD with adjuvant chemotherapy administration was significantly better than those without adjuvant chemotherapy administration (75.4% vs. 42.8% for 5-year OS rate, p < 0.001) and that CSS of patients with colon cancer with the solitary TD with adjuvant chemotherapy administration was significantly better than those without adjuvant chemotherapy administration (82.9% vs. 69.3% for 5-year CSS rate, p < 0.001).

Multivariate Cox survival analyses revealed that OS was significantly associated with T staging (HR = 2.018, 95% CI = 1.545–2.634, p < 0.001; **Table 2**), number of lymph nodes examined (HR = 0.587, 95% CI = 0.434–0.794, p = 0.001; **Table 2**), and age at diagnosis (HR = 1.987, 95% CI = 1.446–2.730, p < 0.001; **Table 2**) in all patients with colon cancer with solitary TD. Moreover, the overall mortality risk of all patients with adjuvant chemotherapy administration was decreased by 64.4% compared with those without adjuvant chemotherapy administration (HR = 0.356, 95% CI = 0.265–0.479, p < 0.001; **Table 2**). Multivariate Cox survival analyses revealed that CSS was significantly associated with T staging (HR = 3.497, 95% CI = 2.430–5.031, p < 0.001; **Table 2**), and number of lymph nodes examined (HR = 0.487, 95% CI = 0.323–0.734, p = 0.001; **Table 2**) in all patients with colon cancer with the solitary TD. Moreover, colon cancer–specific mortality risk of patients with adjuvant chemotherapy administration was decreased by 57.4% compared with those without adjuvant chemotherapy administration (HR = 0.426, 95% CI = 0.286–0.634, p < 0.001; **Table 2**).

**Table 2:** Multivariate Cox regression analysis of prognostic factors for OS and CSS in colon cancer with solitary tumor deposit.

Variable	OS		CSS	
	HR (95% CI)	*P*-value	HR (95% CI)	*P*-value
**T staging**		<0.001		<0.001
** T1–T3**	1		1	
** T4**	2.018 (1.545–2.634)		3.497 (2.430–5.031)	
**Adjuvant chemotherapy**		<0.001		<0.001
** No/unknown**	1		1	
** Yes**	0.356 (0.265–0.479)		0.426 (0.286–0.634)	
**Age at diagnosis (years)**		<0.001		0.864
** ≤65**	1		1	
** >65**	1.987 (1.446–2.730)		1.036 (0.691–1.554)	
**Gender**		0.135		0.967
** Male**	1		1	
** Female**	0.830 (0.650–1.060)		1.008 (0.707–1.435)	
**Tumor location**		0.264		0.544
** Right-sided colon**	1		1	
** Left-sided colon**	0.865 (0.670–1.116)		0.893 (0.619–1.287)	
**Grade**		0.296		0.895
** I/II**	1		1	
** III/IV**	1.176 (0.868–1.593)		0.970 (0.620–1.519)	
**No. of lymph nodes examined**		0.001		0.001
** <12**	1		1	
** ≥12**	0.587 (0.434–0.794)		0.487 (0.323–0.734)	

### Survival Benefit Conferred by Adjuvant Chemotherapy in Patients With T1–T3 Colon Cancer With Solitary Tumor Deposit

**Figure 3:**
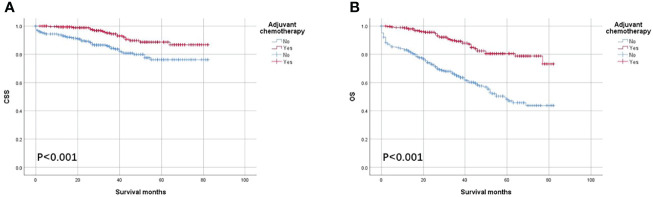
Kaplan–Meier survival curves comparing **(A)** CSS and **(B)** OS according to the use of adjuvant chemotherapy in patients with T1–T3 colon cancer with solitary tumor deposit.

As shown in **Figure 3**, the CSS and OS curves of patients with T1–T3 colon cancer with the solitary TD were generated using the Kaplan–Meier method. It was found that OS of patients with T1–T3 colon cancer with the solitary TD with adjuvant chemotherapy administration was significantly better than those without adjuvant chemotherapy administration (80.5% vs. 48.1% for 5-year OS rate, p < 0.001; **Figure 3A**) and that CSS of patients with colon cancer with the solitary TD with adjuvant chemotherapy administration was significantly better than those without adjuvant chemotherapy administration (88.7% vs. 76.2% for 5-year CSS rate, p < 0.001; **Figure 3B**).

Multivariate Cox survival analyses revealed that OS was significantly associated with age at diagnosis (HR = 2.681, 95% CI = 1.804–3.983, p < 0.001; **Table 3**), and number of lymph nodes examined (HR = 0.602, 95% CI = 0.412–0.879, p = 0.009; **Table 3**) in patients with T1–T3 colon cancer with the solitary TD. Moreover, the overall mortality risk of patients with adjuvant chemotherapy administration was decreased by 65.4% compared with those without adjuvant chemotherapy administration (HR = 0.346, 95% CI = 0.239–0.500, p < 0.001; **Table 3**). Multivariate Cox survival analyses revealed that colon cancer–specific mortality risk of patients with adjuvant chemotherapy administration was decreased by 57.7% compared with those without adjuvant chemotherapy administration (HR = 0.423, 95% CI = 0.246–0.725, p = 0.002; **Table 3**).

**Table 3:** Multivariate Cox regression analysis of prognostic factors for OS and CSS in T1–T3 colon cancer with solitary tumor deposit.

Variable	OS		CSS	
	HR (95% CI)	*P*-value	HR (95% CI)	*P*-value
**Adjuvant chemotherapy**		<0.001		0.002
** No/unknown**	1		1	
** Yes**	0.346 (0.239–0.500)		0.423 (0.246–0.725)	
**Age at diagnosis (years)**		<0.001		0.123
** ≤65**	1		1	
** >65**	2.681 (1.804–3.983)		1.519 (0.893–2.582)	
**Gender**		0.142		0.384
** Male**	1		1	
** Female**	0.800 (0.595–1.077)		0.812 (0.507–1.299)	
**Tumor location**		0.446		0.812
** Right-sided colon**	1		1	
** Left-sided colon**	0.887 (0.652–1.207)		0.946 (0.588–1.524)	
**Grade**		0.081		0.632
** I/II**	1		1	
** III/IV**	1.420 (0.958–2.105)		1.172 (0.612–2.244)	
**No. of lymph nodes examined**		0.009		0.018
** <12**	1		1	
** ≥12**	0.602 (0.412–0.879)		0.509 (0.291–0.889)	

### Survival Benefit Conferred by Adjuvant Chemotherapy in Patients With T4 Colon Cancer With Solitary Tumor Deposit

**Figure 4:**
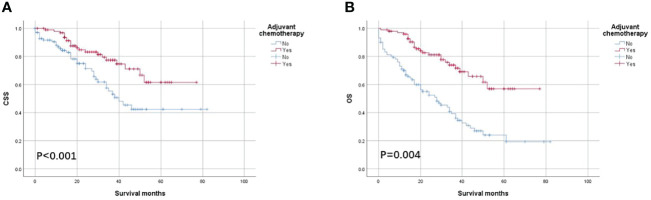
Kaplan–Meier survival curves comparing **(A)** CSS and **(B)** OS according to the use of adjuvant chemotherapy in patients with T4 colon cancer with solitary tumor deposit.

As shown in **Figure 4**, the CSS and OS curves of patients with T4 colon cancer with solitary TD were generated using the Kaplan–Meier method. It was found that OS of patients with T4 colon cancer with the solitary TD with adjuvant chemotherapy administration was significantly better than those without adjuvant chemotherapy administration (56.9% vs. 24.0% for 5-year OS rate, p < 0.001; **Figure 4A**) and that CSS of patients with colon cancer with the solitary TD with adjuvant chemotherapy administration was significantly better than those without adjuvant chemotherapy administration (61.5% vs. 42.3% for 5-year CSS rate, p = 0.004; **Figure 4B**).

Multivariate Cox survival analyses revealed that the overall mortality risk of patients with adjuvant chemotherapy administration was decreased by 68.4% compared with those without adjuvant chemotherapy administration in patients with T4 colon cancer with solitary TD (HR = 0.316, 95% CI = 0.188–0.531, p < 0.001; **Table 4**). In addition, colon cancer–specific mortality risk of patients with adjuvant chemotherapy administration was reduced by 63.3% compared with those without adjuvant chemotherapy administration (HR = 0.367, 95% CI = 0.196–0.686, p = 0.002; **Table 4**).

**Table 4:** Multivariate Cox regression analysis of prognostic factors for OS and CSS in T4 colon cancer with solitary tumor deposit.

Variable	OS		CSS	
	HR (95% CI)	*P*-value	HR (95% CI)	*P-*value
**Adjuvant chemotherapy**		<0.001		0.002
** No/unknown**	1		1	
** Yes**	0.316 (0.188–0.531)		0.367 (0.196–0.686)	
**Age at diagnosis (years)**		0.853		0.062
** ≤65**	1		1	
** >65**	0.947 (0.532–1.686)		0.524 (0.267–1.032)	
**Gender**		0.655		0.225
** Male**	1		1	
** Female**	0.905 (0.585–1.401)		1.419 (0.807–2.497)	
**Tumor location**		0.195		0.298
** Right-sided colon**	1		1	
** Left-sided colon**	0.732 (0.456–1.174)		0.732 (0.406–1.317)	
**Grade**		0.934		0.676
** I/II**	1		1	
** III/IV**	1.020 (0.635–1.639)		0.875 (0.467–1.639)	
**No. of lymph nodes examined**		0.013		0.006
** <12**	1		1	
** ≥12**	0.526 (0.316–0.874)		0.420 (0.227–0.780)	

## Discussion

TD was first described in 1935 and was considered as the deposit of carcinoma cells at a distance from the primary growth (14). This definition has, in fact, changed in colorectal cancer over time. TDs were first introduced in the fifth edition of the The American Joint Committee on Cancer (AJCC)/TNM staging in 1997, where tumor nodules larger than 3 mm in diameter with no histological evidence of residual LN were classified as regional lymph node metastases, whereas tumor nodules smaller than 3 mm were classified in the T category (15).The presence of TD was classified as N1c in the absence of LNM, and the N1c classification was first introduced in the seventh edition of the AJCC/TNM staging to allow us to better understand the clinical significance of TD.

Notably, TD was redefined in the new TNM classification (eighth edition) released in early 2017, and its incidence was reported in previous literature to be between 18% and 25% (12, 16–18). Many previous studies have revealed that the presence of TD was a manifestation of more aggressive tumor biology and should be further investigated by researchers. Gopal et al. reported a trend toward decreased tumor response to neoadjuvant chemoradiotherapy in patients with N1c rectal cancer, which resulted in worse survival and recurrence in this group of patients (19% vs. 10%, p = 0.092 for 5-year local recurrence rate and 3.1 vs. 11.2 years for OS, p = 0.027) (12). Wei et al. found that the presence of TD was an important independent prognostic factor for rectal cancer following preoperative treatment (HR = 2.41, 95% CI =1.24–4.69, p = 0.01) (19). Another study showed that among patients with right-sided colon adenocarcinoma, N1c patients had significantly lower survival compared with N0 patients (20). A higher recurrence rate was also reported in patients with TD-positive colorectal cancer compared with patients with TD-negative colorectal cancer (65.1% vs. 39.1%) (21).

The survival benefit of chemotherapy in patients with colorectal cancer with TD has been inconsistent in previous studies because it was not clear whether the pN1c category was or was not equal to LNM (9). It was reported that the presence and number of TDs did not affect the benefit of chemotherapy in stage III colorectal cancer (22). It has also been shown that patients with TD did not show a benefit in disease-free survival following chemotherapy (23). However, a recent study found that chemotherapy was independently associated with better prognosis in patients with colorectal cancer with TD (HR = 0.542, 95% CI = 0.501–0.586) (24). Despite that, patients with colon cancer with the presence of TD should receive adjuvant chemotherapy after radical surgery according to clinical guidelines for colorectal cancer. Therefore, it is necessary to explore the clinical significance of TD in adjuvant chemotherapy for patients with colon cancer.

Recently, Yeom et al. conducted a retrospective study in 281 patients with colon cancer and found that adjuvant chemotherapy did not provide clear survival benefit for patients with colon cancer with solitary LNM (84.1% vs. 83.3%, p = 0.490 for 5-year disease-free survival). Therefore, it is reasonable to question the survival benefit of adjuvant chemotherapy in patients with colon cancer with solitary TD. In this study, it was found that adjuvant chemotherapy administration could significantly improve OS (75.4% vs. 42.8% for 5-year OS rate, p < 0.001) and CSS (82.9% vs. 69.3% for 5-year CSS rate, p < 0.001) in all patients with colon cancer with the solitary TD. Multivariate Cox survival analyses also showed that adjuvant chemotherapy administration was a good prognosis factor: The overall mortality risk of all patients with adjuvant chemotherapy administration was decreased by 64.4% compared with those without adjuvant chemotherapy administration, and colon cancer–specific mortality risk of patients with adjuvant chemotherapy administration was decreased by 57.4% compared with those without adjuvant chemotherapy administration. The survival benefit conferred by adjuvant chemotherapy in patients with colon cancer with solitary TD in our study was better than survival benefit conferred by chemotherapy in patients with colorectal cancer with TD as reported by Chen et al. (24).

Considering that adjuvant chemotherapy had definite survival benefit in T4 colon cancer, we then performed subgroup analyses according to T stage, and all patients were divided into T1–T3 and T4 subgroups (25–27). Again, subgroup analyses showed that adjuvant chemotherapy had obvious survival benefit in both patients with T1–T3 and T4 colon cancer with solitary TD, which further confirmed the above results. In patients with T1–T3 colon cancer with solitary TD, the overall and colon cancer–specific mortality risks of patients with adjuvant chemotherapy administration were decreased by 65.4% (HR = 0.346, 95% CI = 0.239–0.500, p < 0.001) and 57.7% (HR = 0.423, 95% CI = 0.246–0.725, p = 0.002) compared with those without adjuvant chemotherapy administration, respectively; in patients with T4 colon cancer with solitary TD, the overall and colon cancer–specific mortality risks of patients with adjuvant chemotherapy administration were decreased by 68.4% (HR = 0.316, 95% CI = 0.188–0.531, p < 0.001) and 63.3% (HR = 0.367, 95% CI = 0.196–0.686, p = 0.002) compared with those without adjuvant chemotherapy administration, respectively.

Taken together, our study suggests that adjuvant chemotherapy is warranted and has an obvious survival benefit in patients with colon cancer with the solitary TD. To our knowledge, this is the first time that the efficacy of adjuvant chemotherapy in patients with colon cancer with the solitary TD is proposed and explored. We screened the target population from more than 150,000 patients with colon cancer and performed this retrospective analysis, which provided a high quality of evidence for the use of adjuvant chemotherapy in patients with colon cancer with solitary TD and gave us a better understanding of the clinical significance of TD in colon cancer.

However, our study has some shortcomings. First, this study has the potential selection bias because of the nature of retrospective studies. Second, the patients included in the study have a long longitudinal span from 2010 to 2016, which may have a potential impact on the results of the analysis. Finally, because of database limitations, some important tumor or patient characteristics that may affect the prognosis of patients with colon cancer were not analyzed in this study. In particular, this cancer database did not capture information on specific chemotherapy regimens, so we did not analyze the effect of a particular chemotherapy regimen on the prognosis of patients with colon cancer, which should be deemed as one limitation of the present study. Therefore, the efficacy of adjuvant chemotherapy in patients with colon cancer with the solitary TD still deserves special attention from researchers in the future.

## Conclusions

Adjuvant chemotherapy administration could significantly improve OS and CSS in patients with colon cancer with the solitary TD. This is the first study to investigate and demonstrate the survival benefit of adjuvant chemotherapy in patients with colon cancer with the solitary TD.

## Data Availability Statement

The raw data supporting the conclusions of this article are available from the corresponding author on reasonable request.

## Author Contributions

QL and WY conceived the project and wrote the manuscript. QL, SS, and JL collected the data. WY, HZ, and SS performed the data analyses. All authors contributed to the article and approved the submitted version.

## Conflict of Interest

The authors declare that the research was conducted in the absence of any commercial or financial relationships that could be construed as a potential conflict of interest.

## Publisher’s Note

All claims expressed in this article are solely those of the authors and do not necessarily represent those of their affiliated organizations, or those of the publisher, the editors and the reviewers. Any product that may be evaluated in this article, or claim that may be made by its manufacturer, is not guaranteed or endorsed by the publisher.
